# Supplementary material for: Smartphone-Based Maternal Education for the Complementary Feeding of Undernourished Children Under 3 Years of Age in Food-Secure Communities: Randomised Controlled Trial in Urmia, Iran
Source: Nutrients. 2020 Feb 24;12(2):587. doi: 10.3390/nu12020587 (PMC7071370; doi:10.3390/nu12020587)
Supplement: Supplementary file 1 [file nutrients-12-00587-s001.pdf]

# Smartphone-based maternal education directed at complementary feeding versus treatment as usual among malnourished children below 3 years of age in food-secure contexts: Randomized controlled trial in Urmia, Iran

**Supplement file A.** Study questionnaire.

## STUDY QUESTIONNAIRE

### NUTRITIONAL HEALTH LITERACY SURVEY INSTRUMENT

## SECTION 1- SOCIO- DEMOGRAPHICS

**1- Mother birth date**: **13 --- / --- / ---**

**2- Highest level of education:**

☐ **Elementary school** ☐ **Middle school** ☐ **Diploma** ☐ **Academic**

**3- Mother occupation:**

☐ **House wife** ☐ **Employed at home** ☐ **Employed out of home**

**4- Parents house ownership status:**

☐**Own home** ☐**Leased home**

**5- Car ownership status:**

☐ **Has** ☐ **Has not**

**6- Number of the rooms:**

☐ **Less than 2** ☐ **2 or 3** ☐ **More than three**

**7- Residency:**

☐ **Village** ☐ **City margin** ☐ **City**

**8- Age of the children sequentially:**

**1. --------** **2. -------** **3. --------**

**9- Malnourished child birth date:** **13--- / --- / ---**

**10- Number of the children: ------------**

**11- Child sex:**

☐ **Girl** ☐ **Boy**

## SECTION 2- QUESTIONS for MOTHERS KNOWLEDGE

| | Questions | Yes | No |
|---|---|---|---|
| 1 | Breast milk is more easily digested than formula | | |
| 2 | Breastfeeding does not affect in decreasing post-pregnancy body weight | | |
| 3 | Breastfeeding is helpful in achieving mother's pre-pregnancy womb size faster | | |
| 4 | Mother milk and formula milk health benefits are the same | | |
| 5 | Mother milk has all the essential nutrients for Newborn babies | | |
| 6 | Colostrum does not contain all the essential antibodies to help the child's immune system | | |
| 7 | The best time to start breastfeeding is within one hour of delivery | | |
| 8 | Exclusive breastfeeding to infants in the first 6 month of life is essential | | |
| 9 | The water used to quench the child's thirst in less than 6 month of age, should be boiled | | |
| 10 | The child under the age of six months, needs breastfeeding every three hours | | |
| 11 | Breastfeeding to child under 6 month of age is done when the child is crying | | |
| 12 | Breastfeeding promote mother- baby bonding | | |
| 13 | Mother who practiced breastfeeding has a low risk of getting breast cancer | | |
| 14 | Not all mothers can have sufficient and nutrient milk | | |
| 15 | Breastfeeding should not be continued despite the nipple scarring | | |
| 16 | Mothers do not eat enough, could not produce enough milk | | |

| | | | |
|---|---|---|---|
| 17 | **Only children aged 3 to 9 months should receive iron supplementation** | | |
| 18 | **The use of iron drop causes blackening of the teeth in infants** | | |
| 19 | **Lentils and beans provide needed iron for infants** | | |
| 20 | **At 9-11 months, if your baby does not use breast milk, should give him 1 to 2 additional meals a day** | | |
| 21 | **In the first six months of life, the most nutritional needs of the infant are provided from supplementary food** | | |
| 22 | **From 1 to 2 years old, breastfeeding should be limited to the child** | | |
| 23 | **From 1 to 2 years old, the child should have three to four food meals a day** | | |
| 24 | **From 1 to 2 years old, a variety of sweet drinks can also be used for the baby** | | |
| 25 | **From the age of 2 to 3, four meals a day should be given to the baby.** | | |
| 26 | **From the age of 2 to 3, a snack should be given twice a day between the main meals** | | |
| 27 | **Canned food can be used to prepare baby food.** | | |
| 28 | **It is necessary to refrain from giving honey to the infant** | | |
| 29 | **Avoid giving cow milk until the age of three.** | | |
| 30 | **It is better to use non-pasteurized dairy products (such as yogurt and cheese) to feed the baby.** | | |

## SECTION 3- QUESTIONS for MOTHERS ATTITUDE

| | Questions | Strongly Agree | Agree | Neutral | Disagree | Strongly Disagree |
|---|---|---|---|---|---|---|

| | | | | | | |
|---|---|---|---|---|---|---|
| **1** | **Benefits of breastfeeding are limited for a specific period.** | | | | | |
| **2** | **Bottles are the best option for feeding children with formula milk.** | | | | | |
| **3** | **Mothers with smaller breast size cannot produce enough milk** | | | | | |
| **4** | **Formula-fed babies are more likely to gain weight quickly than breast-fed infants.** | | | | | |
| **5** | **Formula-fed babies are healthier than formula-fed infants.** | | | | | |
| **6** | **Breast milk is the ideal food for babies in the first 6 month of life.** | | | | | |
| **7** | **Breastfeeding is more acceptable than formula feeding.** | | | | | |
| **8** | **Breastfeeding can strengthen the correlation between mother and infant.** | | | | | |
| **9** | **If the mother intends to come back her work, formula feeding is better choice.** | | | | | |
| **10** | **Mothers who formula-fed their children will lose their mother-child bonding experiences** | | | | | |
| **11** | **Mothers who breastfeed babies, fathers will feel isolated.** | | | | | |
| **12** | **If there is enough meat in the diet, use of iron supplementation is not important** | | | | | |
| **13** | **A 6-12 month old baby can use pasteurized milk** | | | | | |
| **14** | **By starting other food meals, child doesn't need breastfeeding any more.** | | | | | |
| **15** | **The child should only use solid food after teeth has started developing.** | | | | | |

| | | | | | | |
|---|---|---|---|---|---|---|
| 16 | **homemade foods are better than Packaged foods** | | | | | |
| 16 | **Packaged foods are nutritious than homemade foods** | | | | | |
| 17 | **Infants should eat from meals prepared for the household only at scheduled times.** | | | | | |
| 18 | **It is not requisite to wash hands before feeding a child.** | | | | | |

## SECTION 4- QUESTIONS for MOTHERS PRACTICE

### Relating to cares for 3 to 5 days until the end of six months

**1- Is the infant repeatedly breastfed at least 8 times a day, according to his demand?**

☐**Yes** ☐**No**

**2- If he does not wake up, you will wake him up after three hours?**

☐**Yes** ☐**No**

**3- Do you breastfeed the infant at nights?**

☐**Yes** ☐**No**

**4- Does breastfeeding continue until complete breast discharge and abandonment by the infant?**

☐**Yes** ☐**No**

5- **Do you give your baby a pacifier?**

☐**Yes** ☐**No**

6- **Do you give any food or fluid other than breast milk to the infant?**

☐**Yes** ☐**No**

## Relating to cares for the end of 6 months until the end of 8 months

7- **Do you prepare your baby food at a suitable density?**

☐**Yes** ☐**No**

8- **Have thickened nymphs and perfectly softened foods that fit the age and nutrient status of child containing a variety of food groups (especially animal protein sources such as meat (sheep and chicken) , fruits and vegetables rich in vitamin A and vitamin C) given to your child?**

☐**Yes** ☐**No**

9- **Do you also give fluids?**

☐**Yes** ☐**No**

10- **When did you stop breastfeeding / or will you? ----------------month of age**

11- **Have you started the supplementary food?**

☐**Yes, please continue the questions**

☐**No, please refer to question 24**

12- **When did you introduce a supplement to your child?**

13- **When did you introduce your child to the following table?**

| **Food item** | **Child Age** | | | | | |
|---|---|---|---|---|---|---|
| | < 3 month | 3- 5 months | 6 months | 6 months- 1 year | After 1 year | Not introduced |
| **Cereals (Rice, bread...)** | | | | | | |
| **sweet potatoes and nymphs** | | | | | | |
| **Fruits** | | | | | | |
| **Vegetables** | | | | | | |
| **Eggs** | | | | | | |
| **Fish/ Meat** | | | | | | |
| **Skimmed/ low fat milk** | | | | | | |
| **Cow milk** | | | | | | |
| **Tea, sweets, chocolates** | | | | | | |

**14- How often do you feed your child?**

**I. 6 to 8 months : A) < 2 times B)2 to 3 times C)3 to 5 times D) >5 times**
**II. 9 to 12 months : A) <3 times B)3 to 4 times C)4 to 6 times D) >6 times**
**III. > 12 months : A) <3 times B)3 to 4 times C)4 to 6 times D) >6 times**

**15- Which food group you gave your child the day or the last night (please mark)?**

| **Food** | |
|---|---|
| Rice, bread, pasta, maize, Soft and watery feed | ☐ |
| sweet potatoes and nymphs | ☐ |
| Banana, oranges, mango, pineapple, water-melon | ☐ |
| Tomato, onion, green pepper | ☐ |
| Fish, meat, birds meat | ☐ |
| Milk, cheese, yogurt, eggs | ☐ |
| Cerelac, or other ready foods | ☐ |

| | |
|---|---|
| Breast milk | ☐ |
| Fruit juice, flavored water | ☐ |
| Tea, coffee | ☐ |

**16- Do you add salt to your baby's food? ☐Yes ☐No**

**17- By which of the following do you feed your baby?**

**A) Feeding bottle B) Bowl and spoon C) hand feeding**

**18- Do you wash your hands before feeding your child? ☐Yes ☐No**

**19- Do you wash and sterilize baby food containers after use? ☐Yes ☐No**

**20- How do you feed the sick child when has lost appetite?**

**A) slowly and patiently B) By favorite foods C) Forcefully**

**21- Why haven't introduced complementary feeding?**

**A) The child does not have the necessary age**

**B) Breast milk alone is enough**

**C) Insufficient fund D) Other items.....**
